# A hybrid hydrogel encapsulating human umbilical cord mesenchymal stem cells enhances diabetic wound healing

**DOI:** 10.1007/s10856-022-06681-4

**Published:** 2022-07-18

**Authors:** Hongjie Xu, Jingjing Wang, Di Wu, Dajiang Qin

**Affiliations:** 1 Innovation Centre for Advanced Interdisciplinary Medicine, Key Laboratory of Biological Targeting Diagnosis, Therapy and Rehabilitation of Guangdong Higher Education Institutes, The Fifth Affiliated Hospital of Guangzhou Medical University, Guangzhou, 510799 China; 2 Department of Neurology, Weihai Central Hospital, Weihai, China; 3 Bioland Laboratory (Guangzhou Regenerative Medicine and Health Guangdong Laboratory), Guangzhou, 510663 China; 4 Present Address: Bioland Laboratory (Guangzhou Regenerative Medicine and Health Guangdong Laboratory), Guangzhou, 510663 China; 5 Present Address: Innovation Centre for Advanced Interdisciplinary Medicine, Key Laboratory of Biological Targeting Diagnosis, Therapy and Rehabilitation of Guangdong Higher Education Institutes, The Fifth Affiliated Hospital of Guangzhou Medical University, Guangzhou, 510799 China

## Abstract

**Background:**

Diabetic wound is a severe complication of diabetes. Stem cell is considered as a promising therapy for diabetic skin wounds. Hydrogel can supply niche for cells adhesion and survival to improve the efficacy of stem cell therapy, but the development of hydrogel with suitable properties remains a great challenge. Thus, our study was conducted to combine an optimized hydrogel with stem cell to improve complex diabetic wound treatment.

**Methods:**

This study constructed a hydrogel with low toxicity and adjustable mechanical properties from gelatin methacrylate (GelMA) and chitosan-catechol (Chi-C), and encapsulated human umbilical cord-mesenchymal stem cells (hUMSCs) to repair full-thickness diabetic wound.

**Results:**

We explored the relationship between mechanical stiffness and cell proliferation and differentiation potency, and found 10% GelMA hydrogel with an optimal stiffness improved hUMSCs adhesion, proliferation, and differentiation potency maintenance in vitro. Assistant with optimized hydrogel encapsulating hUMSCs, diabetic wound healing process was greatly accelerated, including accelerated wound closure, inhibited secretion of inflammatory factors TNF-α and IL-1β, promoted vascular regeneration and collagen deposition after treatment of hUMSCs.

**Conclusions:**

The optimized hydrogel encapsulating hUMSCs improved diabetic wound healing, and has a broad implication for the treatment of diabetic complication.

**Figure:**
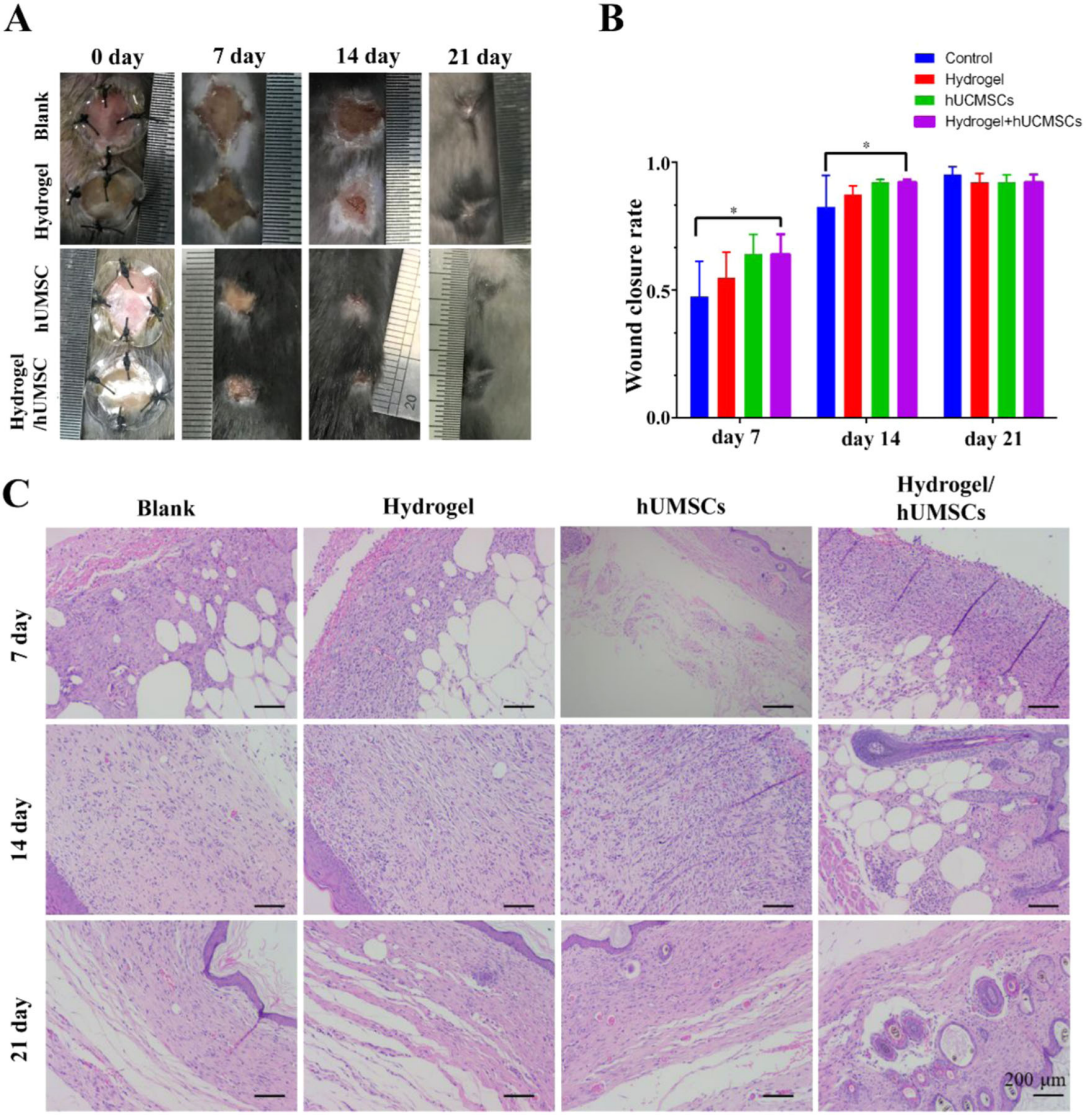
Diabetic wound is a severe complication of diabetes. Stem cell is considered as a promising therapy for diabetic skin wounds. Hydrogel can supply niche for cells adhesion and survival to improve the efficacy of stem cell therapy. This study constructed a hydrogel with low toxicity and adjustable mechanical properties from gelatin methacrylate (GelMA) and chitosan-catechol (Chi-C), and encapsulated human umbilical cord-mesenchymal stem cells (hUMSCs) to repair full-thickness diabetic wound. Hydrogel of 10% GelMA with an optimal stiffness improved hUMSCs adhesion, proliferation, and differentiation potency maintenance in vitro. Assistant with optimized hydrogel encapsulating hUMSCs, diabetic wound healing process was greatly accelerated, including accelerated wound closure, inhibited secretion of inflammatory factors TNF-α and IL-1β, promoted vascular regeneration and collagen deposition after treatment of hUMSCs. The study supplies an alternative treatment for diabetic complication. Hydrogel-hUMSCs combined treatment accelerates wound closure in diabetic mice. **A**. Representative images of wounds during 21-day in vivo experiments. **B**. Quantification of wound closure rate (%) over 21-day period. **C**. HE staining of wounds at days 7, 14 and 21. The bar corresponds to 200 μm.

## Introduction

Diabetes is a global health problem in modern societies that has been estimated to affect 439 million people worldwide by 2030 [1]. Diabetic foot ulcer (DFU) is a severe complication of diabetes, which affects quality of life and threatens life [1]. This nonhealing wounds as a serious chronic health problem need more explore for effective approach [2–5]. Regarding to the complicated pathophysiological abnormalities including peripheral neuropathy, persistent inflammation, impaired re-epithelialization, reduced angiogenesis and blood flow, the development of a combined therapeutic approach is essential [6].

Wound healing has synergistic multiple processes including three overlapping phases: inflammatory, proliferative, and remodeling phase [7]. The endogenous mesenchymal stem cells (MSCs) are core coordinator to balance the immune response and tissue rebuilding by recruiting immune cells and secreting immune factors, growth factors and matrix proteins [8]. However, the endogenous MSCs of diabetic patients have various functional abnormality like hyperphophorylation of insulin receptor substrate-1 (IRS-1) and excessive expression of early growth response factor-1 (EGR-1) and its target genes (PTEN and GGPS1) [9, 10]. In recent years, it has been a promising process for stem cells-based therapy to repair cutaneous wounds [1, 11–16]. Human umbilical cord-mesenchymal stem cells (hUMSCs) have been reported as a promising source, for their abundant sources, high self-renewal properties and low immunogenicity [17–19]. HUMSCs have powerful effectiveness for wound repair in bone, brain, heart and so on [20]. HUMSCs therapy was found remarkable effect for vascularization, re-epithelization and blood flow without side-effect [21, 22]. HUMSCs implantation accelerates cutaneous wound healing in diabetic rats via the Wnt signaling pathway [23]. However, the effect of stem cells-based therapies is impaired because of adhesion difficulty and high cell death rate for harsh environment characteristics of severe diabetic ulcers. To protect the stem cells and maintain their activity, a combined therapeutic approach with advanced materials is of great promise.

Hydrogel has been widely used in the three-dimensional culture of stem cells due to its three-dimensional network structure mimicking extracellular matrix. They can also improve the retention and survival of transplant stem cells in the hostile wound niche [24–26], hence promote wound healing. The promotion effect of MSCs derived from bone marrow or adipose could be enhanced by hydrogels, such as multi-acrylated poly (ethylene glycol) macromers and thiolated hyaluronic acid, dextran-hyaluronic acid, Pluronic F-127, chitosan (CS), fibrin, methacrylated gelatin (GelMA) and methacrylated hyaluronic acid and so on [25, 27–31]. These hydrogels promoted wound closure, inhibited inflammation for repairing non-healing wound like diabetic wound and burn [27, 29–33]. Furthermore, they showed ability to increase collagen deposition, cytokines secretion and tissue remodeling, providing a potential treatment for high-quality wound closure and efficient scar inhibition [30, 34].

Many materials could induce remarkable neovascularization and skin regeneration depending on their good biocompatibility and mechanical properties [29, 30, 35–39]. One of the factors that affects the differentiation behavior of stem cells is the stiffness of the hydrogel. The mechanical stiffness of different tissues in the human body varies greatly, which is usually less than 10 kPa in the soft tissues. In order to promote the differentiation of stem cells in skin tissues, we have prepared hydrogels with different mechanical stiffness to study the in vivo repair effects of stem cells. Gelatin contains a large amount of RGD peptides, which has good promoting effect on cell adhesion. Gelatin methacrylate (GelMA) is a chemically modified product of gelatin. It has adjustable cross-linking rate and mechanical strength, and has huge application prospects in the field of biological 3D printing. Catechol-modified chitosan (Chi-C) has shown good hemostatic effects, water solubility and cell adhesion ability [40]. In this study, we used dithiothreitol (DTT) to crosslink GelMA and Chi-C, formed a hydrogel through a mild Michael addition reaction, and encapsulated mesenchymal stem cells in the hydrogel during the gel formation process. In addition, zinc ions were introduced into the hydrogel to enhance vascularization. The mass concentration of GelMA was changed to prepare hydrogel with different mechanical stiffness through changing the crosslinking density. HUMSCs morphology, adhesion, proliferation and differentiation were evaluated in hydrogel with different mechanical stiffness. HUMSCs encapsulated in the hydrogel with optimized mechanical stiffness were selected and further tested for repairing diabetic wound in mice.

## Materials and methods

### Materials

Gelatin (type A, from porcine skin) and chitosan (low molecular weight) was purchased from Sigma-Aldrich (Shanghai, China). Methacrylic anhydride was purchased from Aladdin (Shanghai, China). 3,4-Dihydroxyhydrocinnamic acid, N-(3-dimethylaminopropyl)-N′-ethylcarbodiimide hydrochloride (EDC), ethanol (AR, 99.7%), DL-dithiothreitol (DTT) and zinc chloride (ZnCl_2_) were purchased from Macklin (Shanghai, China). CCK-8 proliferation assay kit was purchased from Beyotime (Shanghai, China). Dulbecco’s modified Eagle’s medium/Ham’s F-12 medium (DMEM/F-12), induction media (osteogenic, chondrogenic and adipogenic) and fetal bovine serum (FBS) were ordered from Invitrogen. Cell culture plate was ordered from Corning.

### Synthesis of gelatin methacrylate (GelMA) and chitosan-catechol (Chi-C)

#### Synthesis of GelMA

1.0 g of gelatin was fully dissolved in PBS buffer under magnetic stirring at 50 °C to obtain a transparent liquid. Then 0.6 mL of methacrylic anhydride was added dropwise to the gelatin solution. The whole reaction was kept for total 1 h at 50 °C, after that the reaction solution was transferred to a dialysis bag (molecule weight cutoff, MWCO = 3500 Da) and dialyzed against deionized water at 40 °C for 3 days to remove the by-product. The final product was obtained by lyophilization and stored at −20 °C until use.

#### Synthesis of Chi-C

The chitosan-catechol (Chi-C) was synthesized according to previous methods [40]. 0.5 g of chitosan was fully dissolved in 0.1 M HCl under stirring to obtain 1% (w/v) chitosan solution and the pH value was adjusted to 4~5 using 1 M NaOH solution. Then 0.29 g of 3,4-dihydroxyhydrocinnamic acid (HCA) and 0.62 g of EDC were separately dissolved in 3 mL of deionized water and 50 mL of water/ethanol (v:v = 1:1) solution. Mix the HCA and EDC solution and pour onto the chitosan solution immediately. The reaction was kept for 18 h with the pH value around 4~5. The reaction solution was dialyzed against deionized water (pH = 5) and molecule weight cut off was 3500 Da. The final product was obtained by lyophilization and stored at −20 °C until use.

### Preparation of hydrogel with different mechanical strength

The hydrogel with different mechanical strength was obtained by adjust the concentration of GelMA. Table 1 shows the concentration of different components in the hydrogel. Take the first group as an example, firstly, the GelMA and Chi-C was separately dissolved in suitable PBS. Subsequently, the two solutions were mixed, and the pre-prepared ZnCl_2_ solution and DTT solution were immediately added to the above mixed solution. After fully mixing, the solution was stand at room temperature until the hydrogel was completely formed.

**Table 1:** The concentration (m/m) of different component in the hydrogel

	Chi-C	GelMA	DTT (mg/mL)	ZnCl_2_ (mM)
Group I	2%	6%	20	20
Group II	2%	10%	20	20
Group III	2%	14%	20	20

### Characterization of the hydrogel

The chemical structure of GelMA and Chi-C were characterized by proton nuclear magnetic resonance (^1^H NMR, Bruker, ACSEND^TM^ 600, Germany). D_2_O was used as solvent for the ^1^H NMR testing. The morphology of the lyophilized hydrogel was characterized by scanning electron microscope (SEM, Philips, XL-30, Netherlands) and the samples were sprayed with gold before observation. Mechanical strength of the hydrogel was measured by universal testing machine (Bose, ELF3200, America). Cylindrical samples (ϕ = 11 mm, height = 6 mm) were prepared and kept at moist environment until testing. The strain was set to 60% and compression speed was 0.5 mm/s. Rheological behavior of the hydrogel was tested by rotational rheometer (Malvern, Kinexus Pro, the UK). Time sweep sequence was tested at fixed stress (1 Hz) of 25 °C. Frequency sweep sequence was tested at fixed strain (1%) with a stress changed from 0.1 Hz to 10 Hz.

### In vitro degradation behavior of the hydrogel

The in vitro degradation behavior was obtained by measure the mass of the hydrogel. Cylindrical hydrogel samples were firstly weighted and the mass of the hydrogel at initial status was recorded as M_0_. Then the hydrogel was totally immersed in PBS buffer and placed at 37 °C. Take out the hydrogel at pre-set time points and gently wipe out the water on the surface. The weight of the hydrogel at different time point was recorded as M_t_. The degradation ratio was finally expressed as the hydrogel weight ratio calculated by the following equation:

$${{{\mathrm{Hydrogel}}}}\,{{{\mathrm{weight}}}}\,{{{\mathrm{ratio}}}} = {{{\mathrm{M}}}}_t/{{{\mathrm{M}}}}_0 \times 100\%$$

### Toxicity analysis of hydrogels

Cytotoxicity tests of hydrogels were detected using CCK-8 assay, as previously described [41]. The hydrogels with different concentration of GelMA were soaked in DMEM/F-12 medium supplemented 10% FBS in a 37 °C incubator for 24 h. The supernatant was collected, and filter sterilized as 100% hydrogel extracts. HUMSCs was cultured in extracts for 1 day, 3 days or 7 days 96 well plates. Extract was refreshed containing 100 μL 10% CCK8 solution, incubated 2 h in a dark environment. The absorbance of each sample at 450 nm was then read immediately from the microplate reader (GENios; TECAN, Atlanta, GA, USA).

### Adhesion and differentiation of hUMSCs on hydrogels

HUMSCs were supplied by the Lanri biotechnology co., LTD as reported [42]. Briefly, Wharton’s jelly was teased out of the cord and collected in another Petri dish, and the arterial blood vessel, venous blood vessel, and amnion were discarded. The Wharton’s jelly was sliced into small fragments around 1 mm in diameter and added to culture flasks till cells migration. Cells were proliferated using DMEM/F-12 medium supplemented with 10% FBS in an incubator at 37 °C and 5% CO_2_. The cells were identified by flow cytometry. Briefly, hUMSCs were incubated with fluorescence-conjugated specific monoclonal antibodies against CD73 (BV771), CD90 (APC/cy7), CD105 (BV421), CD34 (PE), CD45 (FITC), CD14 (AF700), HLA-DR (APC) for 30 min at 4 °C in the dark. After staining and washing, cells were acquired using a BD LSR-Fortessa X-20 flow cytometer (BD Biosciences) and analyzed with Flowjo software.

To explore the adhesion and differentiation ability on the hydrogels of different mechanical stiffness, hUMSCs were grafted on gradient GelMA hydrogels or non-hydrogel as control group and induced by three induction media separately, then observed and stained by oil red O, Alizarin red, and alcian blue to detect the morphology.

### Wounding protocol and closure analysis

24 diabetic mice (*db/db*) were purchased from Chinese Academy of Medical Science & Peking Union Medical College Institute of Biomedical Engineering. Two round-shaped (8 mm diameter) incisions penetrating the subcutaneous tissue were created on the back of anesthetized each mouse using a pair of scissors. Two incisions of every mouse were performed using two treatments to inject 100 μL PBS or hUMSCs, hydrogel or hydrogel-hUMSCs treatment. HUMSCs amount was 5 × 10^6^. Cells were injected directly or mixed with solution before hydrogel formation and transplanted hydrogel. The wound closure was analyzed after 7, 14 and 21 days. Exposed areas of the wounds were obtained using Image J software (NIH, Bethesda, MD, USA). Wound closure rate (%) was defined as (origin wound area -residual wound area at day ‘x’)/origin wound area ×100%.

### Histological analysis

Wound tissues were collected and then fixed in 4% formaldehyde overnight at scheduled time points. All tissues were dehydrated in a graded series of ethanol, and embedded in paraffin. Tissues were sectioned into slices with a thickness of 5 μm and for routine haematoxylin-eosin (H&E, Sigma-Aldrich) staining, Masson’s trichrome staining (Sigma-Aldrich) and visualized by an optical microscope.

### Immunohistochemistry (IHC) and immunofluorescence (IF)

For IHC and IF to analyze inflammation and vascularization, slides were deparaffinized and then rinsed in PBS for 5 min and 3 times. Then blocked with 5% serum for 25 min and followed incubated in primary antibodies, TNF-α (1:300), IL-1β (1:300), VEGF (1:100), CD31 (1:300) and α-SMA (1:50). The nuclei were stained with DAPI. The stained sections were observed using an optical microscope (BO-M30; AOSVI, Shenzhen, China) or a fluorescence microscope (Nikon, Melville, NY).

### Statistical analysis

Statistical analysis was performed with GraphPad Prism 6 program. Differences with a value of *p < 0.05 were considered statistically significant after analyzed with Student’s *T* test when the data was normally distributed. All values are expressed as the mean ± standard deviation of at least three replicates.

## Results

### Characterization of mechanical adjustable hydrogels

**Fig. 1:**
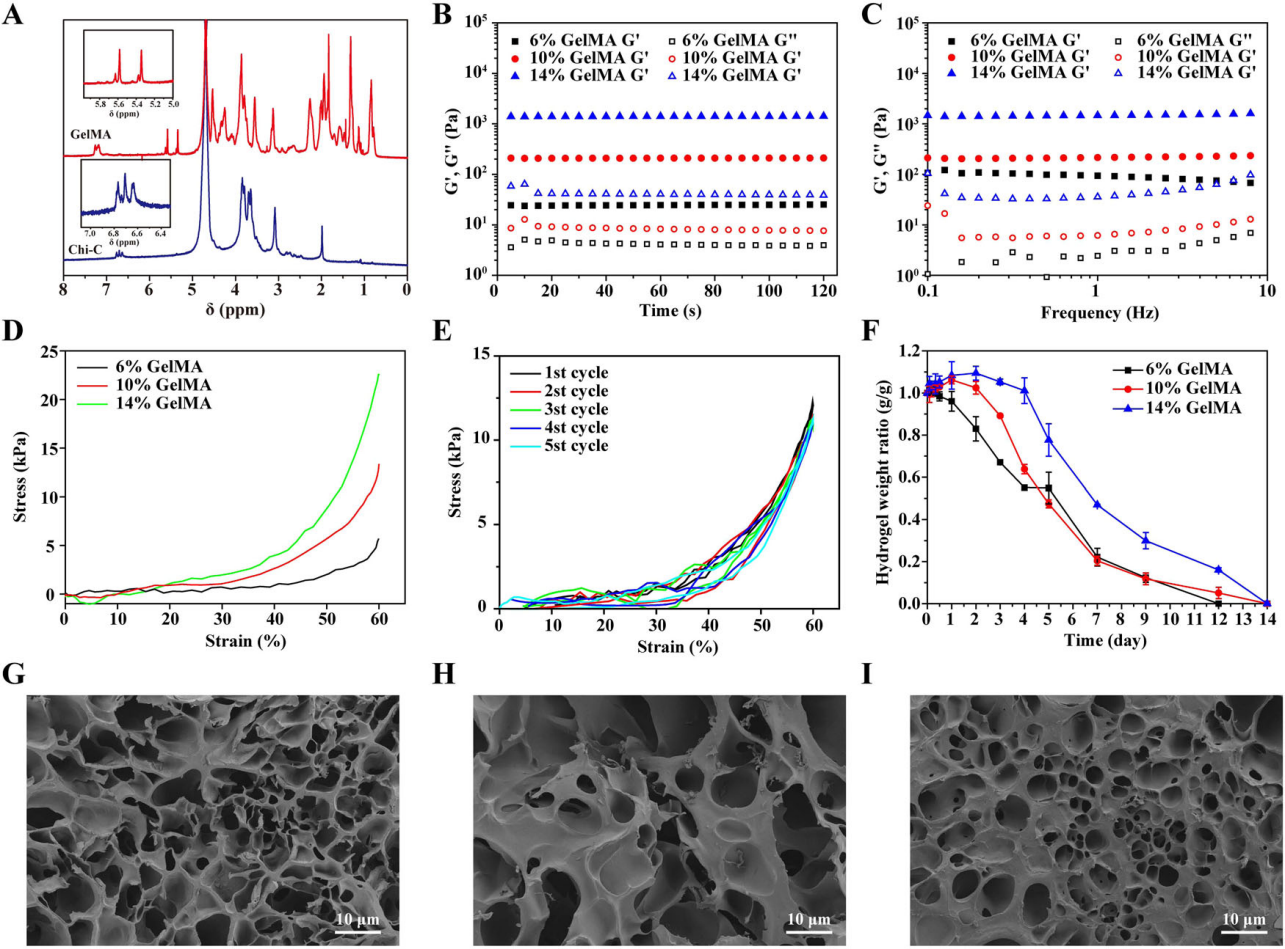
Characterization of the hydrogel with different GelMA concentration (6%, 10%, 14%). **A** ^1^H NMR spectrum of GelMA and Chi-C. **B** Time sweep sequence and **C** frequency sweep sequence of hydrogel. **D** Static compression test of hydrogel. Cyclic compression test of the hydrogel. **E** In vitro degradation behavior. **G**–**I** Morphology of lyophilized hydrogel with GelMA concentration of 6%, 10% and 14%

Mechanical strength is one of the important factors regulating stem cell behavior. In order to obtain mechanical adjustable hydrogel to regulate stem cell fate and achieve the best diabetic skin damage treatment effect, we prepared three different hydrogels with increased mechanical strength. ^1^H NMR spectrum (Fig. 1A) showed the successful synthesis of GelMA and Chi-C (GelMA: ^1^H NMR [D_2_O], δ [ppm]: 5.58, 5.34 (GelMA, -C = CH_2_; Chi-C: ^1^H NMR [D2O], δ [ppm]: 1.99 (Chi-C, -COCH_3_), 6.65, 6.71, 6.79 (Chi-C, aromatic ring proton)). The GelMA and Chi-C were crosslinked by dithiothreitol (DTT) at room temperature. Rheological and mechanical test were conducted after the hydrogel was totally formed. As shown in Fig. 1B, as the mass concentration of GelMA increased, the storage moduli (G’) of GelMA/Chi-C hydrogel were 24.48 Pa, 209.7 Pa and 1399 Pa, respectively. The difference of G’ was attributed to the increased crosslinking density of hydrogel. More C-S bonds were formed between GelMA thus leading to the stiffer hydrogel. Frequency sweep sequence showed that the GelMA/Chi-C hydrogels were in the linear viscoelastic region within the range of 0.1–10 Hz and showed good elastic behavior (Fig. 1C).

A similar tendency was also shown in the compression test. The GelMA/Chi-C hydrogel containing 14% GelMA had the highest compressive stress at 60% strain, reaching 22.54 kPa. While the GelMA/Chi-C hydrogel containing 10% GelMA and 6% GelMA were 13.34 kPa and 5.71 kPa, respectively (Fig. 1D). The cyclic compression curve of 10% GelMA/Chi-C hydrogel was shown in Fig. 1E. There was no significant energy loss during the 5 cycles. It showed that the GelMA/Chi-C hydrogel could resist certain elastic deformation. This property is of great significance for wound dressings. It can resist the deformation of the skin during exercise without breaking, thus can significantly improve the comfort of the user.

The degradation behavior of hydrogels is also an important factor affecting stem cell proliferation and migration. The pores produced during the degradation of the hydrogel can promote the in-growth of stem cells, and facilitate the neovascularization and tissue growth. Figure 1F showed that the GelMA/Chi-C hydrogel could fully degrade in vitro during 14 days. The hydrogel with higher GelMA concentration first showed a certain swelling performance, began to degrade on the third day, and then completely degraded in 14 days. It was worth noting that the 6% GelMA hydrogel began to degrade slowly on the first day, and completely degraded after 12 days. But there was no significant swelling behavior, which was not conducive to the absorption of wound exudate, so this proportion of hydrogel may not be conducive to the repair of wound tissue. This hypothesis will be verified in vivo.

The SEM images of the three hydrogels with different mechanical strengths we prepared after freeze-drying showed regular pore diameter changes (Fig. 1G–I). The higher the GelMA content, the greater the mechanical strength of the hydrogel and the smaller the corresponding pore size. This hydrogel with different mechanical strength and pore size will be used as a stem cell carrier to study the differentiation behavior of hUMSCs and its repair effect on diabetic wound.

### hUMSCs isolation and characterization

**Fig. 2:**
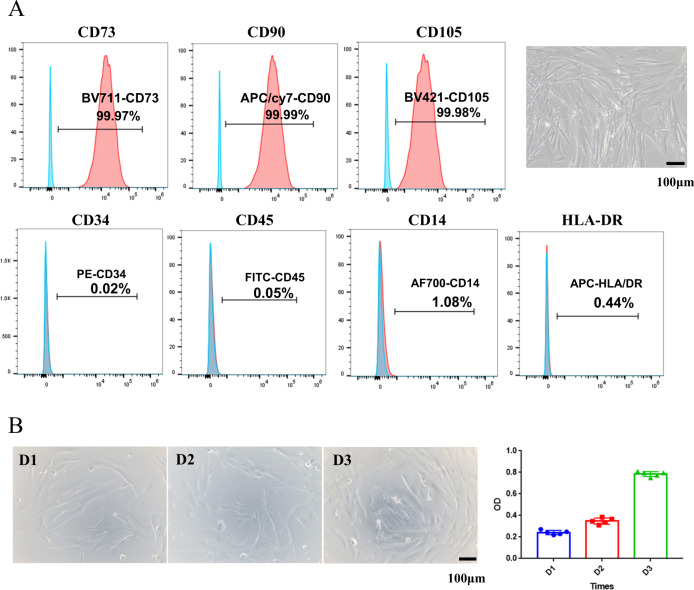
Immunophenotypical analysis of hUMSCs. **A** HUMSCs were labeled with specific antibodies for the indicated MSCs (upper panels) or hematopoietic (bottom panels) markers and analyzed by flow cytometry. **B** Evaluation of hUMSCs proliferation at 1st, 2nd, 3rd day

Human mesenchymal stem cells were isolated from umbilical cord as previously reported [41]. Flow cytometry analysis of hUMSCs demonstrated that the expression of mesenchymal stem cells surface markers CD73, CD90 and CD105 were greater than 99.9% (Fig. 2A, upper panels), while the expression of hematopoietic markers CD34, CD45, CD14, and MHC-class II HLA-DR were barely detectable (less than 1.1%, Fig. 2A, bottom panels). The seeded hUMSCs also showed significant proliferation on 3th days (Fig. 2B). These results suggested that hUMSCs with high quality and purity were successfully acquired.

### Biocompatibility of hydrogels

**Fig. 3:**
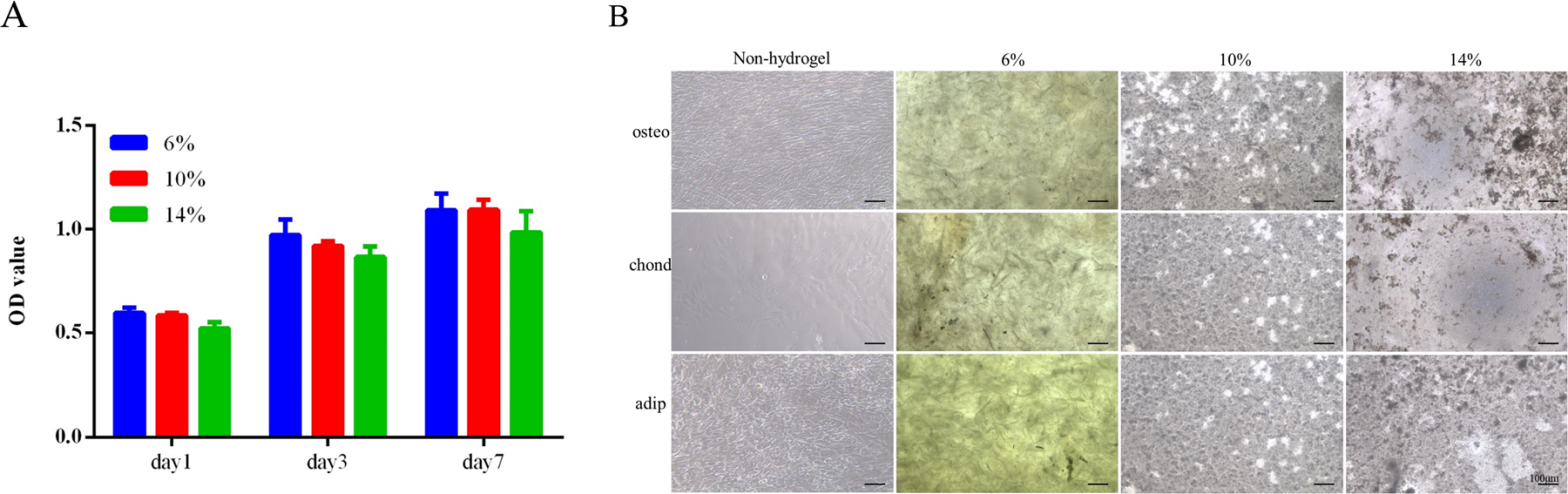
Biocompatibility of hydrogels. **A** CCk-8 assay of hUMSCs culture in leach liquor of three hydrogels or medium as control for 1, 3 and 7 days. **B** Cellular morphology of hUMSCs culture on hydrogels or tissue culture plate in inducing media for 14 days

To determine the biocompatibility of hydrogels, proliferation test was performed for hUMSCs with leach liquor from different concentration of GelMA. CCK-8 assay showed similar cell proliferation rate between leach liquor and normal medium and no significant difference among leach liquor from different concentration of GelMA after culture for 1, 3, 7 days (Fig. 3A).

To confirm the effect of hydrogels on hUMSCs proliferation and differentiation, hydrogels with different stiffness were used to culture hUMSCs in differentiation inducing medium. We observed that the cellular morphology of the hUMSCs on hydrogels had remarkable three-dimensional characteristics compared with that cultured on tissue culture plate without hydrogel after 14 days. More important, only 10% GelMA hydrogel had confluent cells with the highest density, in fact thick cellular lays totally different from the monolayer on tissue culture plate, while the other two hydrogels only a few colonies (Fig. 3B). The result clearly demonstrated the importance of mechanical stiffness to cell adhesion.

These results suggested diversity response of stem cells to different proportions of hydrogel with different mechanical characteristics. Hydrogels changed stem cells growth pattern totally. It has been reported the material content, pore size, stiffness and nanostructure affect stem cell adhesion and cell fate choice via mechanotransduction, because hydrogel as extracellular matrix interact directly with cell surface protein, such as integrins which connect with cytoskeleton to regulate cell morphology and signal transduction [42–51].

### Differentiation of hUMSCs on hydrogels

**Fig. 4:**
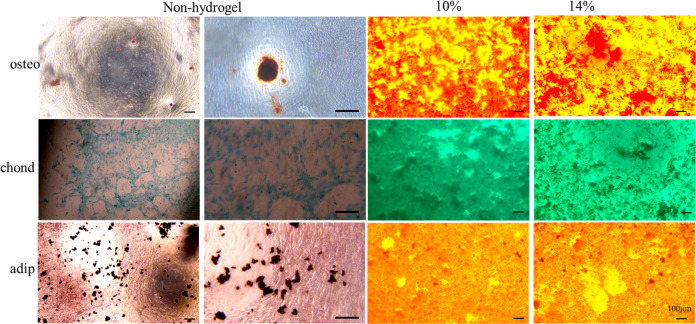
Differentiation of hUMSCs on hydrogels. Staining images of hUMSCs culture on hydrogels or tissue culture plate in inducing media (osteoblastic, chondroblast and adipose differentiation) for 21 days

To determine differentiation potential of hUMSCs on hydrogels and fate determination of the stem cells under different mechanical characteristics, we induced osteogenesis, cartilage and adipogenic differentiation on three hydrogels for 21 days and executed staining of alizarin red, alcian blue and oil red O, respectively. The assay showed similar positive colour of hUMSCs on 10% and 14% GelMA hydrogels compare with non-hydrogel control. Interestingly, hUMSCs cultured on hydrogels, especially 10% GelMA hydrogel, had higher density than that on tissue culture plate (Fig. 4). It suggested that hydrogel with suitable mechanical properties not only maintained stemness of hUMSCs but also reserved more functional cells, which may improve the performance of stem cell for wound repair in vivo. Our results were consistent with previous reports indicating the effect of hydrogels on stem cells differentiation and cell function were regulated by mechanical properties [52–54]. Based on this, we chose the 10% GelMA hydrogel as optimized performance for follow-up experiment.

### Hydrogel-hUMSCs combination promoted wound healing

To analyze the efficiency of the hydrogels for acceleration wound closure, a humanized excisional full-thickness skin wound model was developed in the leptin receptor deficient (*db/db*) diabetic mice, which can better mimic DFU pathological mechanism than drug induced diabetic mice.

**Fig. 5:**
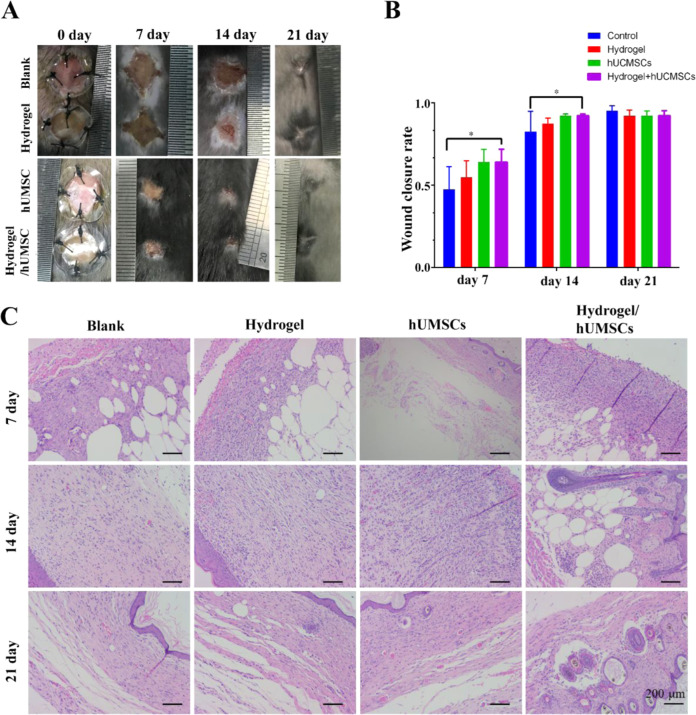
Hydrogel-hUMSCs combined treatment accelerates wound closure in diabetic mice. **A** Representative images of wounds during 21-day in vivo experiments. **B** Quantification of wound closure rate (%) over 21-day period. **C** HE staining of wounds at days 7, 14 and 21. The bar corresponds to 200 μm

Wounds treated with hydrogel/hUMSCs showed significantly accelerated wound closure rate than the control treatment over 7 days (64.1% and 42.8%, *p* < 0.05) and 14 days (92.2% and 78.7%, *p* < 0.05), slightly higher than hydrogel or cell only treated wounds. All the wounds were close to healing and the differences were normalized after 21 days (Fig. 5A, B). HE staining further confirmed the aforementioned result at all the three time points. It is worth mentioning that the epithelioid tissue structure has been observed at 7 days, which appeared till 14 days in stem cells only treatment group (Fig. 5C). These results indicated hydrogel-hUMSCs combined treatment promoted the wound healing process.

### Hydrogel-hUMSCs combination dampened inflammation

To further confirm the improvement of hydrogel combined hUMSCs on diabetic wounds healing and to explore the mechanism, next, we conducted immunohistochemistry and immunofluorescence to examine inflammatory factors, revasculatization and re-epithelization.

**Fig. 6:**
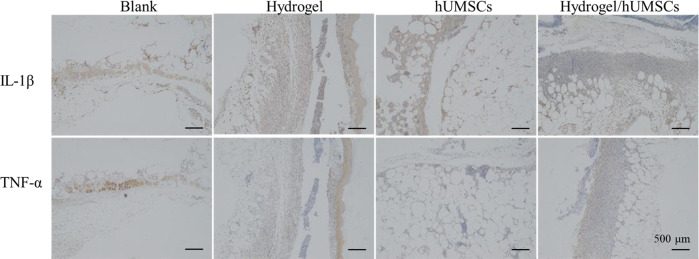
Immune factors expression of wounds for day 7. Expression of TNF-α at the wound sites of rats in the control, the hydrogel and hUMSCs alone or combined group at 7 days after the operation, as revealed by IHC. The bar corresponds to 500 μm

Inflammation is the first one in the three phases of wound healing. Excessive inflammatory response severely hinder diabetic wounds healing. It has been reported that hUMSCs injections dampened rat myocardial fibrosis and dysfunction via inhibiting inflammatory factor TNF-α [20]. The primary role of TNF is regulating immune cells and immune factors secretion, like IL-1 and IL-6. We examined TNF-α and IL-1β protein level after 7 days using IHC and showed that stem cells individual or combined treatment inhibited both protein expression throughout the wounds (Fig. 6). The result also revealed that integrity of organizational structure in any treatment group was better than that in control group.

### Hydrogel-hUMSCs accelerated angiogenesis and re-epithelialization

**Fig. 7:**
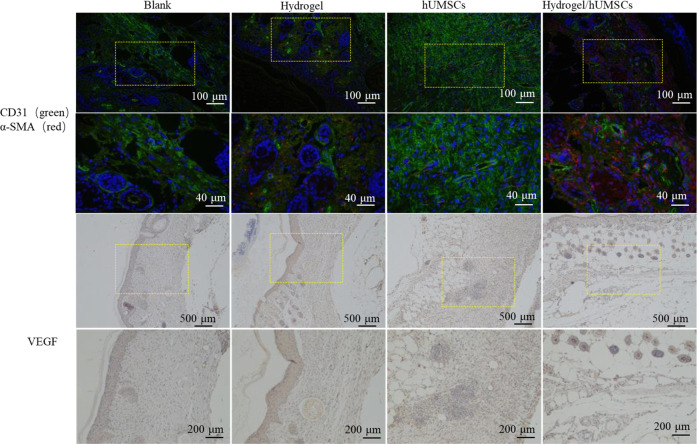
Hydrogel-hUMSCs combined treatment promotes angiogenesis and re-epithelialization. Expression of CD31, α-SMA and VEGF at the wound sites of rats in the control, the hydrogel and hUMSCs alone or combined group 14 days after the operation, detected by immunofluorescence or IHC. High magnification corresponds to boxed area in the low-magnification images

Angiogenesis and re-epithelialization are critical processes in the proliferative phase, the second phase of wound healing. Hallmark proteins related to vascular development (namely CD31, α-SMA and VEGF) were detected. Immunofluorescence assay displayed the number of α-SMA and CD31 double positive stained vessels were highest in the hydrogel/hUMSCs treatment group on day 14, though the α-SMA positive vessels in hUMSCs only treated group was also abundant (Fig. 7). The histological analysis showed that tissues treated with hydrogel/hUMSCs had most remarkable VEGF expression which was clearly distributed around the blood vessel, while only fuzzy outline were observed in hUMSCs treated group and others on 14th day (Fig. 7).

Consistently, we found vascular structure only formed in hUMSCs and hydrogel/hUMSCs treatment group after 7 days. We observed the blood vessels through the skin samples in hydrogel/hUMSCs treatment group after 7 days and in hUMSCs treatment group after 14 days. More complex tissue structure like hair follicle and mature blood vessel also appeared at the earliest in hydrogel/hUMSCs treatment group on 14th and 21st day, respectively (Fig. 5C).

Vascularization has been known as a challenge of DFUs. In our previous study, we have constructed novel hydrogels which combined hUMSCs, and suggested that promoted more blood flow than stem cells or hydrogel individual treatment after 3, 7 and 14 days in rat wounds [41]. Excitingly, the optimized chitosan-based hydrogel united with hUMSCs in this study contributed to observable blood vessel formation after 7 days. Moreover, epidermis-like cell arrangement first appeared in the hUMSCs group and the combined treatment group on 7^th^ day, coinciding with our previous study [41]. What’s more, cells proliferation around the wound in the hUMSCs individual group and the hydrogel-hUMSCs combined treatment group were both more active than the other two groups before 21 days (Fig. 5C). Intriguingly, the formation of hair follicle around the wound was found only in the hydrogel-hUMSCs combined treatment group on 14th day (Fig. 5C).

These data demonstrated that the hydrogel of appropriate mechanical stiffness could assist efficiency of hUMSCs on microcirculation and re-epithelialization, and contribute to diabetic wound closure, even induce hair follicle regeneration. Dextran hydrogel and MSCs was proved respectively to promote dermal regeneration with complete skin appendages [36, 55]. These studies identify a clinical evaluation method for managing lower limb ischemia and rat acute skin wound in our previous study [22, 41, 56].

### Hydrogel-hUMSCs promoted collagen deposition

**Fig. 8:**
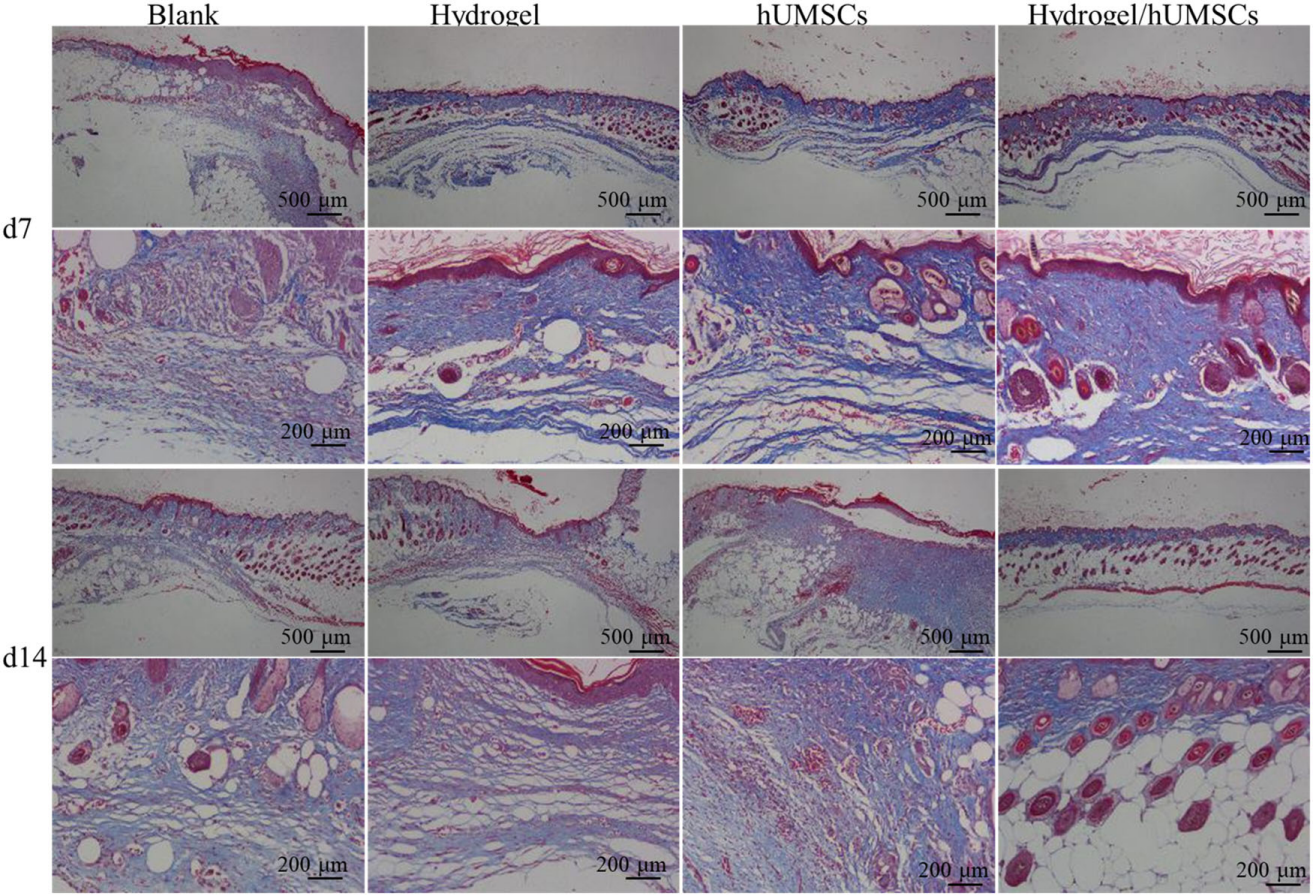
Masson’s trichrome staining of wound sections at day 7 or 14 post-wounding for four conditions

The last phase of wound healing is tissue remodeling phase. It has been reported that hydrogel-MSCs combination enhance fibroblast proliferation in dermis, at the same time, the proliferated fibroblast will start to secrete ECM, the majority of which is collagen [57]. We identified collagen mainly deposited in and around the wound on 7th day in all the groups other than the control, moreover, the hUMSCs individual or combined groups had visualized significant abundance (Fig. 8). However, the collagen deposition was most widely distributed in the hydrogel/hUMSCs treated group. On 14th day, collagen deposition was distributed around the wound in individual treated groups but only aggregated in epidermis, which suggested the collagen had been degraded (Fig. 8). The result demonstrated that stem cells promoted collagen deposition and hydrogel further accelerated the process, therefore improved the tissue remodeling and wound healing. It was consistent with the above results and the recent research [34].

## Discussion

So far, the synthesis and application of GelMA and Chi-C composites has few studies. Additionally, potential of such composites for regulating stem cells function to promote nonhealing wound repair has not been studied. Here, we successful synthesized and characterized the structural properties of GelMA/Chi-C hydrogels. We also seeded hUMSCs on hydrogels and examined the cell adhesion, proliferation and differentiation. Then efficiency of hydrogels encapsulated hUMSCs of promoting diabetic mice wound repair was evaluated. Such MSCs containing hydrogel wound be valuable in stimulating revascularization and skin regeneration in poorly vascularized chronic wounds-a growing burden for patients and healthcare providers worldwide [58].

The study mainly found that GelMA/Chi-C hydrogel affect the hUMSCs adhesion and proliferation dependent on mechanical properties, and that the optimized hydrogel could support hUMSCs in maintaining stemness and in promoting diabetic wounds repair.

DFU is a chronic complication of diabetes characterized by continuity, repeatability, and nonhealing. In recent years, perinatal period mesenchymal stem cells hydrogel complex has been a new emerging technique in the treatment of DFU [59]. We improved some technical details to get hUMSCs with outstanding performance (patent licensed) (Fig. 2). They could be passaged 30 times and maintain the fundamental cell function.

The previous study has revealed that GelMA hydrogels displayed minor cytotoxicity on hUMSCs and umbilical vein endothelial cells (HUVECs), significantly enhanced both cells proliferation, ECM deposition and angiogenic genes expression in vitro [13]. GelMA and methacrylated hyaluronic acid (HAMA) hydrogels loaded with adipose derived stem cells (ADSC) showed the desired proliferative and angiogenic properties essential to promote angiogenesis for wound healing [31]. GelMA/Chi-C hydrogel here also has similar effect on hUMSCs proliferation and differentiation but we furtherly found, the effect dependent on the GelMA concentration (Figs. 3 and 4). In other word materials function depends on its mechanical properties. It proved that cells behavior regulated by materials mechanical properties or stimuli [51, 60–62]. Ours optimized GelMA/Chi-C hydrogel has medium elasticity coefficient which in line with the property of skin tissue. It enhanced angiogenesis of diabetic wound in vivo (Fig. 5). Sufficient attention should be paid to this in the material design in the future. Many studies supply the good references [63, 64].

Besides the stiffness, the pore size of three-dimensional porous scaffolds modulates the behaviors of mesenchymal stem cells [49, 50]. The optimized hydrogel has the proper pore size. Of course, the stiffness and pore size probably are bounded. Few literatures studied these two parameters separately, maybe because of limited methods to unbound these two elements. The hydrogel could fully degrade in vitro during 14 days (Fig. 1). During the time, diabetic wound could heal if hUMSCs carried by hydrogel were transplanted. The rate of hydrogel degradation is appropriate for it acting as a dermal substitute to carry stem cells for wound repair. Hence, the hydrogel has excellent characteristics to promote wound healing.

To confirm the efficiency in vivo, we examined the three overlapping phases: inflammatory, proliferative, and remodeling phase [7] during wound healing. Results showed hUMSCs encapsulated with optimized hydrogel inhibited inflammation (Fig. 6), promoted cells proliferation and angiogenesis (Fig. 5) and accelerated collagen deposition in the last tissue remodeling phase (Fig. 8). Stem cells functions probably involved the whole process of wound heal. Promoting wound repair of stem cells was accelerated by optimized hydrogel, especially reflected by the blood flow and angiopoiesis (Figs. 5 and 7). It’s important to the diabetic wound repair. The problem of blood vessels is the key to heal diabetic wound. MSCs recruited to the injured secreted large amounts of growth factors including epidermal growth factor, fibroblast growth factor, hepatocyte growth factor, and vascular endothelial growth factor for anti-inflammation [65]. The repair of blood vessel function contributed to growth factors and nutrition transportation. This niche rebuilding is getting more and more attention. We focus this and are exploring new hydrogels to enhance the adaptation of stem cells transplanted to hostile environment of nonhealing wound. However, considering the difference of animals and human, more studies that hUMSCs and hydrogel composites transplanted to human DFU are necessary. More clarity of molecular mechanism is also worth of study.

## Conclusions

In summary, we have developed a hydrogel with adjustable mechanical property to construct stem cell delivery platform for diabetic wound healing. The hydrogel with optimized mechanical stiffness showed high bio-compatibility and maintain the hUMSCs adhesion, proliferation, survival and stemness in vitro. The therapy system promoted the wound closure, inhibited inflammation, accelerated the vascular regeneration and deposition of collagen in vivo, and eventually improved the diabetic wound healing. Together, this study described a novel alternative approach to treat non-healing wound.

## Data Availability

The primary data for this study is available from the authors on direct request.

## Abbreviations

GelMA: gelatin methacrylate
Chi-C: chitosan-catechol
hUMSCs: human umbilical cord-mesenchymal stem cells
DFUs: Diabetic foot ulcers
MSCs: mesenchymal stem cells
IRS-1: insulin receptor substrate-1
EGR-1: excessive expression of early growth response factor-1
hUMSCs: human umbilical cord-mesenchymal stem cells
CS: chitosan
DTT: dithiothreitol
HUVECs: umbilical vein endothelial cells
HAMA: methacrylated hyaluronic acid
ADSC: adipose derived stem cells
